# Impacts of low-head hydropower plants on cyprinid-dominated fish assemblages in Lithuanian rivers

**DOI:** 10.1038/s41598-020-78701-8

**Published:** 2020-12-10

**Authors:** Tomas Virbickas, Paolo Vezza, Jūratė Kriaučiūnienė, Vytautas Akstinas, Diana Šarauskienė, Andrius Steponėnas

**Affiliations:** 1 Nature Research Centre, Akademijos 2, 08412 Vilnius, Lithuania; 2 Department of Environment, Land and Infrastructure Engineering, Politecnico di Torino, Corso Duca degli Abruzzi, 24, 10129 Turin, Italy; 3 Lithuanian Energy Institute, Breslaujos 3, 44403 Kaunas, Lithuania

**Keywords:** Ecology, Hydrology

## Abstract

The meso-scale habitat simulation model MesoHABSIM was applied in three Lithuanian lowland rivers to study the effect of low-head hydropower plants (HPPs) on the fish habitats. Stream flow time series on a daily scale for the period 1970–2015 were used to describe flow regime downstream of HPPs for periods before and after their installation. Conditional habitat suitability criteria were developed for 4 species of cyprinid fish, schneider (*Alburnoides bipunctatus*), dace (*Leuciscus leuciscus*), roach (*Rutilus rutilus*) and vimba (*Vimba vimba*) to simulate their available habitat at different water discharges. Modelling results showed that HPPs have a significant impact on habitat availability in the low flow period in dry years below HPPs due to insufficient released flow. The environmental flow, as prescribed by the Lithuanian national law, is estimated between 80 and 95% exceedance probability of the mean minimum discharge of 30 days. This flow leads to a significant reduction in frequency and duration of available suitable habitats for vimba and schneider during low flow period. The roach habitat is the least affected. The results of habitat modelling are in line with the actual data on the occurrence and relative abundance of considered fish species in the studied river stretches. A general comparison of the relative abundance of modelled fish species in 42 natural river stretches and 20 stretches below the HPPs also showed that the relative abundance of roach is significantly higher, and that of schneider is significantly lower in river sections below the HPPs than the abundance in natural river sections. All results indicate that the current environmental flow does not secure survival of certain fish species. The applicability of the average low flow release during summer could be a plausible alternative to the current environmental flow in order to maintain ecosystem health and services.

## Introduction

The interruptions of the longitudinal continuity of rivers, changes in river hydromorphology, hydro-peaking and variation of flow regime can have multiple impacts on fish, including changes to physical habitat, habitat access, food supplies, behaviour, community composition, energy expenditure, and population dynamics^1^. Poff and Zimmerman^2^ reviewed 165 papers looking for a relationship between various kinds of flow alteration and ecosystem responses. The vast majority of these scientific studies reported decreasing values for the analysed ecological metrics in response to hydrological alterations. Even small run-of-river hydropower plants (HPPs) can have significant ecological impacts under certain conditions^3^. There is a growing body of literature that recognises the importance of environmental principles in the management of regulated rivers^4–6^. It was demonstrated that small changes in water flow regimes in the management of dams can help to restore river ecosystems^7^. The European Water Framework Directive^8^ CIS guidance document No 31^9^ provides a common understanding of what is meant by the ecological flow (e-flow): "amount of water required for the aquatic ecosystem to continue to thrive and provide the services we rely upon". Ecological flow is widely used as an HPP management measure to balance human and aquatic ecosystem needs, since the determined e-flow should guarantee suitable conditions for the existence of aquatic communities. Nowadays, there are many models related to water ecosystems and physical environment on a micro-, meso- and macro-habitat scale developed to estimate environmental flows and requiring a different amount of expert knowledge, data and finances^10^.

According to the general criteria described by Abbasi and Abbasi^3^, all hydropower plants in Lithuania, except the largest Kaunas HPP, are small HPPs operating in the run-of-river (RoR) mode (low-head HPPs with energy output up to 2.9 MW). Almost all of the HPPs (97%) return the diverted flow directly below the dam; it is a common belief that RoR HPPs do not affect the amount of water in the downstream section (i.e. hydrological alteration can be considered negligible). In the current Lithuanian national law related to planning, use and maintenance of HPPs, the environmental flow (Q_env_) prescription below HPPs is estimated between 80 and 95% exceedance probability of the mean minimum discharge of 30 consecutive days^11^. Environmental restrictions are also imposed on HPP reservoir water level variation, which should be no more than ± 10 cm of normal water level. There are no other restrictions in force for HPPs. Consequently, in low flow periods, low-head HPPs may increase the frequency and duration of Q_env_ in downstream river sections. According to the river discharge projections^12^, in future, dry periods are expected to become more frequent, i.e. the current Q_env_ will continue to be released downstream the HPP for even a longer period. Although it is officially considered that the established environmental discharge provides minimum conditions for ecosystem survival, there is still no scientific basis for this.

In general, there is a lack of studies for low-head facilities operating in temperate lowland rivers^13^. Hydropower production is generally concentrated in mountainous areas due to favourable topography and larger water availability^14^. For low-head HPPs, many studies in literature mention that the major impact on fish communities is due to obstacles for migration, while the impact of hydrological alteration in low flow periods has been weakly studied^15,16^. In Lithuania, some studies focusing on the impact of HPPs on the hydrological regime have been made. Alterations of the hydrologic regime were examined and downstream river flow (stage) ramping was identified using the recorded hourly data of flow/stage downstream power plants^13^. To reduce the effects of river flow ramping, simple turbine operational measures, such as step-wise turbine start-up and shut-down together with varying turbine numbers and capacities over 24 h, were proposed. Several studies of the effect of small HPP dams on the ecological status of invertebrate assemblages revealed a decline in the number of taxa and the total abundance of benthic macroinvertebrates both upstream and downstream of dams compared to control sites^17,18^. However, the impact of HPPs at different flows and the effects on organisms other than benthic invertebrates have not yet been studied. The ecological effectiveness of environmental flow release has also never been evaluated. Therefore, the aim of this study was to assess whether hydrological alterations caused by the functioning of low-head HPPs can have significant impacts on fish habitats in lowland rivers of Lithuania in comparison with natural hydrological regime (without HPP activity). In addition, the study aims to evaluate whether Q_env_, which is currently guaranteed in regulated river sections, provides suitable conditions for the maintenance of fish communities.

## Study area

The country's territory (65.3 thou. km^2^) is located below 215 m above sea level and is drained by four main river basins (RBs): Nemunas, Venta, Lielupe and Daugava. All these rivers flow into the central part of the eastern coast of the Baltic Sea and belong to biogeographic region of Central Europe that shows the lowest fish species endemism^19^. Differences in climatic conditions within the country are small; therefore, the main ecological factors in structuring the communities of river fish are the area of the catchment, which determines the total number of species, and the slope of the channel, which determines the presence of indicator species and the composition of ecological guilds. Salmonids dominate only in small (< 100 km^2^ catchment area) watercourses with a predominant supply of groundwater. Larger rivers are dominated by cyprinids, and salmonids are found only in stretches with a higher slope of the channel^20^.

**Figure 1:**
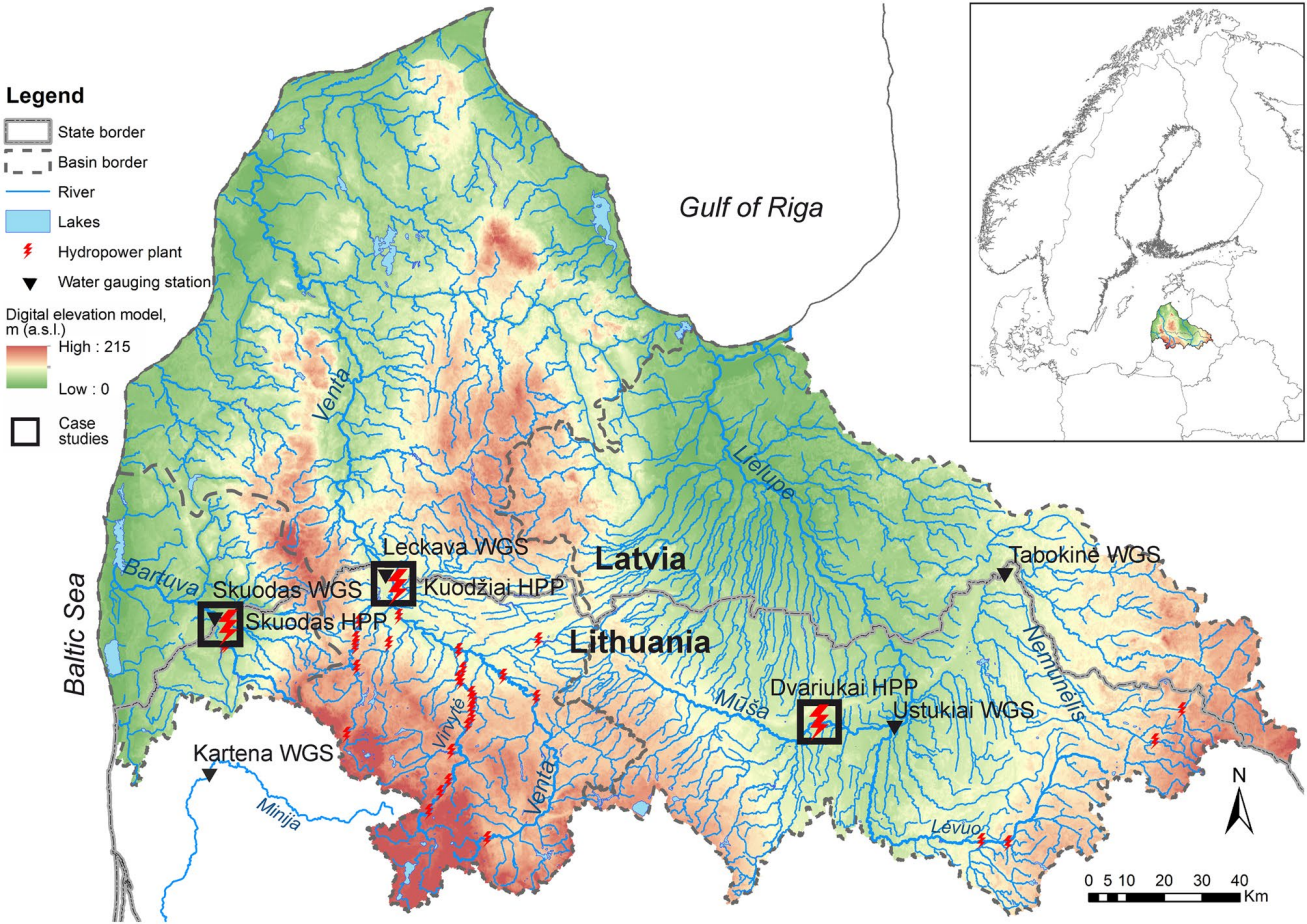
Study area and location of the selected case studies.

As case studies, three rivers (Bartuva, Venta and Mūša) from two main RBs (Venta and Lielupe) were selected (Fig. 1). The local climatic conditions of selected rivers mostly differ in the precipitation amount. The highest annual precipitation amount (750–850 mm) is observed over the Bartuva River catchment. Whereas the Mūša River catchment falls within the area of less than 650 mm of annual precipitation. 60% of precipitation coincides with the warm season, but due to increased evaporation (460–540 mm), only a small part of them transforms into river discharge (except flash flood event). The selected rivers have predominant surface feeding; therefore, the absence of strong groundwater supply determines high flow variability during the year and highly expressed low-flows during the warm season. All rivers can be classified as transboundary rivers, since their RBs are located in both Lithuania and Latvia. The overall area of the Venta and the Lielupe river basins is around 33.2 thous. km^2^. The Venta and the Lielupe RBs are significantly affected by low-head HPPs. In total, 30 HPPs are constructed on the Venta RB and 5 HPPs on the Lielupe RB. As an example of application, three low-head HPPs that return the diverted flow directly below the dam, namely Skuodas, Kuodžiai and Dvariukai, located on the Bartuva, Venta and Mūša rivers, respectively, were selected as case studies (Fig. 1). The main HPP and RB characteristics of the three case studies are listed in Table 1. The installed capacity of the selected HPPs varies in the range from 220 to 600 kW.

**Table 1:** Characteristics of the selected case studies at the particular hydropower plant (HPP).

	Case study 1	Case study 2	Case study 3
River	Bartuva	Venta	Mūša
Water gauging station	Skuodas	Leckava	Ustukiai
HPP	Skuodas	Kuodžiai	Dvariukai
HPP construction year	2000	2005	2001
Distance from the mouth (km)	52.8	188.9	81.5
Catchment area (km^2^)	260	4021	1927
River bed slope (m/km)	0.57	0.61	0.73
Area of reservoir (ha)	85.9	25.3	136.4
Dam height (m)	8.00	4.50	5.80
Installed capacity (kW)	220	600	494
Q_env_ (m^3^/s)^a^	0.220	1.75	0.380
Q_30_min_ (m^3^/s)	0.120	1.22	0.361
Q_30_ave_ (m^3^/s)	0.320	5.25	1.19
Q_30_max_ (m^3^/s)	0.790	12.8	3.57
Q_annual_mean_ (m^3^/s)	3.18	30.3	8.68

^a^Environmental flow (Q_80%_ for Bartuva and Q_95%_ for Venta and Mūša) is derived from the rules of exploitation of HPP reservoirs.

## Methodology

The meso-scale habitat simulation model MesoHABSIM^21^ was used to assess the impact of low-head HPPs on fish populations. MesoHABSIM is a physical habitat modelling system developed for e-flow assessment and river channel restoration planning. It describes the utility of instream habitat conditions for aquatic fauna, allowing to simulate change in habitat quality and quantity in response to alterations of flow and river hydromorphology. Meso-scale habitats are defined as geomorphic units (GUs, such as pools, riflles, rapids, glides^22^) that can be used by species and life stages for a significant part of their diurnal routine^23^. A meso-habitat can be considered suitable or optimal when the configuration of hydraulic patterns, together with the attributes that provide shelter, create favourable conditions for survival and development of animals. MesoHABSIM approach is based on the aggregation of three models^24^:

1. A hydromorphological model that describes the spatial mosaic of fish-relevant hydro-morphological features.
2. A biological model describing the relationship between the presence and abundance of fish and the physical environment of the river.
3. A habitat model quantifying the amounts, frequency and duration of the available habitat depending on the flow regime and local river morphology.

For the modelling, the time series of daily water discharge data in natural and altered (downstream HPPs) conditions were created for wet, normal and dry years in order to describe the habitat suitability in all possible hydrological conditions. Conditional habitat suitability criteria (CHSC) were developed to define the relationship between fish distribution and physical environment. Physical spatial measurements of river hydraulic and fish shelter attributes (current velocity, depth, discharge, sediments, woody debris, boulders, etc.) were conducted on a scale of mesohabitat during field surveys. SimStream plugin of QGIS^25^ was used to organize collected data for mesohabitat modelling.

### Hydrological data and hydromorphological surveys

The daily time series of discharge data of three water gauging stations (WGSs; Bartuva-Skuodas, Venta-Leckava and Mūša-Ustukiai) were taken from the hydrological yearbook of the Lithuanian Hydrometeorological Service for the periods of 1970–2000 (period before construction of HPPs) and of 2001–2015 (period after construction). The WGSs are located downstream the selected HPPs, and their data were used for the assessment of the altered discharge conditions and the impact of HPPs on fish communities. Two additional WGSs of Minija River-Kartena (for the Bartuva and Venta rivers) and Nemunėlis River-Tabokinė (for the Mūša River) were chosen for the restoration of natural conditions of river discharge at case study sites according to the analogy method^26^. The selection of a river analogue was based on the same hydrological region, similar catchment area, similarity in physico-geographical and hydrometeorological characteristics, and absence of anthropogenic structures which interrupt the continuity of the river, e.g. dams. The regression equation between case study river and river-analogue was prepared using daily water discharge data of 1970–2000 (period before construction of HPPs). The natural regime of investigated rivers after construction of HPPs (2001–2015) was restored using regression equations. In this way, we obtain the annual hydrographs of the investigated rivers in natural and altered conditions. In order to evaluate the habitat suitability in all possible hydrological conditions, hydrographs were prepared for wet, normal and dry hydrological years (probability of 5, 50 and 95%, respectively), according to average discharge data in the period of 2001–2015.

Four different discharge values (from minimal to average) were defined for hydromorphological measurements in each site of the selected river. These discharges represented the minimum, average and maximum low flow discharges of 30 consecutive days (Q_30_min_, Q_30_ave_, Q_30_max_) in the warm period (May–September), and multi-annual mean water discharge (Q_annual_mean_) in 1970–2000 (before HPPs construction). According to the Lithuanian law, environmental flow (Q_env_) is defined at each HPP as 80% or 95% probability of the mean minimum discharge of 30 consecutive days of the warm period^11^. A Laser Rangefinder (distance, inclination, azimuthal measurements) connected via Bluetooth with the field tablet was used for the mapping of hydromorphological units (HMUs, also called mesohabitats). The maps of HMUs polygons were digitized in the .shp format using MapStream plugin of QGIS^25,27^. The length of an analysed river reach was defined as 20 times the mean river width^28^. The depth and flow velocity measurements in each defined HMU were done using a propeller-type flow meter mounted on a wading rod. Depending on the polygon area, from 5 to 30 measurements were carried out in each HMU, while the measurement density (point/m^2^) was kept as constant as possible in each case study considering its size (on average one point per 6 m^2^ in the Bartuva, 20 m^2^ in the Mūša and 25 m^2^ in the Venta rivers).

The presence/absence of fish shelters and vegetation were assessed visually (see^21^ for details). All measurements were carried out as close as it is possible to four defined discharges (minimum low flow (Q_30_min_), average low flow (Q_30_ave_), maximum low flow (Q_30_max_) and annual mean (Q_annual_mean_)) of each selected case study (Table 1).

### Fish data and conditional habitat suitability criteria

Four Cyprinidae fish species, which are common in cyprinid-dominated lowland rivers of Lithuania^20^, but differ in rheophily and reproduction habitat were selected for the assessment of HPPs impact: lithophilic rheophilic schneider *Alburnoides bipunctatus* and dace *Leuciscus leuciscus*, phyto-lithophilic eurytopic roach *Rutilus rutilus*, and diadromous lithophilic eurytopic vimba *Vimba vimba* (fish guilds according to^29^). Based on the classification of fish species in European rivers according to their overall resistance to habitat degradation^30^, the selected species also represent different guilds of tolerance capacity: schneider is intolerant species, dace and vimba are intermediate, and roach is tolerant^31^. These four species are all benthopelagic, and in this respect they are similar, but due to their different preferences for rheophilic conditions, spawning habitat and overall habitat quality, it was expected that their response to changes in flow conditions should also be different. Currently access for diadromous vimba to most rivers is limited by dams; therefore, habitat availability for vimba was modelled only in the Venta River, which is still accessible for this species and contains its spawning grounds.

To define conditional habitat suitability criteria (CHSC)^21^, the river monitoring database for 2008–2015 was used. Data on the physical, chemical and hydrological characteristics of river sites was collected by the Lithuanian Environmental Protection Agency (EPA). Fish monitoring and assessment of hydromorphological characteristics of the site at the time of sampling was carried out by the Nature Research Centre under agreement with EPA. Standardized single-pass electric fishing took place in mid-July–September on river sections with a minimum length of at least 10 times the wetted width (but not less than 50 m) using backpack pulse current electrofisher (type IG200-2; HANS GRASSL GmbH) with a maximum output of 800 V and a maximum power of 10.0 kW per pulse.

For CHSC construction, only river sites in natural conditions (from good to high ecological status according to the European Water Framework Directive) with a catchment area of 100–5000 km^2^ and sampled by wading were selected from the database. In total, 245 river sites were selected. 160 sites in 75 rivers (2/3 of the selected sites) were randomly selected and used to build CHSC. The remaining 85 locations in 53 rivers (1/3 of all locations) were used for calibration. Once the locations were selected, their depth and current velocity were classified into intervals of 0.15 m and 0.15 m s^-1^ following the MesoHABSIM protocol (up to 0.15, 0.15–0.3, 0.3–0.45, etc.). The preference of schneider, dace and roach for depth and current velocity was determined by their frequency of occurrence in each of the intervals. In order to minimize the impact of random catches, species were considered present only when the number of individuals exceeded 25th percentile of the number of individuals in all places where they were found. Species were considered abundant when the number of individuals was greater than the median abundance in all places where they were found. A species was considered present in a particular interval of depth or current velocity only when its frequency of occurrence was > 40%. Accordingly, a species was considered abundant only in those groups of depth and velocity where the number of individuals was greater than the median in more than 50% of the sites. The preference for the type of substrate and shelters was determined according to the analysis of these environmental variables in the river sites where the species should be present based on the criteria of depth and current velocity. According to the geomorphological and ecological definition of mesohabitat^21,22^, 10 m^2^ was considered the minimum surface that an HMU must have to be considered a suitable (species present) or optimal (species abundant) habitat for fish. When tested on an independent dataset (85 sites), CHSC were considered satisfactory for the presence of species when the species were present in > 60% of the sites meeting the criteria (total accuracy > 0.6). CHSC were considered satisfactory for the abundance of species when the species were present in > 60% of the sites meeting the abundance criteria and the abundance of individuals was higher than the median in at least 50% of these sites.

CHSC for vimba were selected by an expert judgement, analysing common features of the river sites where this species was observed. Migration of vimba to the majority of former spawning grounds is currently restricted by dams. Therefore, this species is constantly found in a limited number of rivers, in which vimba is present not only during spawning in spring, but is also common in specific habitats in summer and autumn.

For the validation of CHSC for schneider, dace and roach, a single-pass electric fishing was performed in 42 HMUs of 4 natural rivers (Minija, Dubysa, Šventoji and Merkys), in river stretches with a length of 150–400 m, a maximum depth up to 1.5 m, and a catchment size of 315–3040 km^2^, during the low flow season, with high transparency of water. Fish were sampled by wading by a team of 3 persons using a backpack pulse current unit of a similar type as for fish monitoring (IG200-2D; HANS GRASSL GmbH). CHSC verification for vimba was carried out only in 14 out of 42 HMUs, since this species is constantly found in only one of the natural rivers selected for verification. A single-pass electric fishing was also conducted in all HMUs which were identified in the studied river stretches below HPPs at the low flow. Fish sampling was accomplished by wading and using pulse current backpack electric fishing gear. A single-pass electric fishing strategy was used, as the CHSC criteria were also developed based on single-pass sampling data. Studies show that in most cases species composition and rank abundance of common species do not change significantly after the first pass^32–34^.

To assess the predictive performance of CHSC, correctly classified instances, sensitivity, specificity, and true skill statistic were calculated based on confusion matrix analysis^35^.

### Assessment of HPPs impact

The habitat area available for the species was modelled at different discharges of rivers. The impact of HPPs on habitat availability was assessed based on the comparison of the modelled available habitat area (i) at reference conditions during a dry year, (ii) under HPPs functioning in dry, normal and wet years, and (iii) at environmental Q_env_. The flow value that exceeded 97% of the time at reference conditions (Q_97_)^36^ during a dry year and the corresponding area of species habitat (expressed in m^2^, hereafter, the minimum threshold area) were used as common denominators. Deviation of temporal availability of suitable habitats for modelled fish species due to HPPs functioning at different flows was assessed based on relative increase in the cumulative continuous duration of days when the area of the habitat falls below the minimum threshold values (hereafter, the stress days alteration; SDA). SDA analysis is based on the assumption that minimum habitat availability is a limiting factor for fish species, and events occurring rarely in nature create stress to aquatic fauna and shape the community. Therefore, for the selected minimum habitat threshold (expressed in m^2^), the number of habitat stress days that occur under those conditions was calculated and used as a benchmark for comparative analysis using the SDA metric, (see e.g.^28,36,37^ for details). Finally, we normalize SDA values between 0 and 1 by using the index of temporal habitat availability (ITH) as it is described by Rinaldi et al.^28^.

The relative abundance of fish species that are common in the cyprinid-dominated rivers of Lithuania (the frequency of occurrence in the natural river sites is > 50%) was also compared in river reaches with natural (42 sites, 85 fishing occasions) and regulated (below HPPs; 20 sites, 39 fishing occasions) flows, which met at least good water quality criteria and fell within the same range of catchment size and slope as the rivers selected for modelling did. The sites were selected from the same river monitoring database for 2008–2015, which was used for selection of sites for CHSC development. The significance of identified differences was assessed using the Mann–Whitney U test.

## Results

### Analysis of hydromorphological data

**Figure 2:**
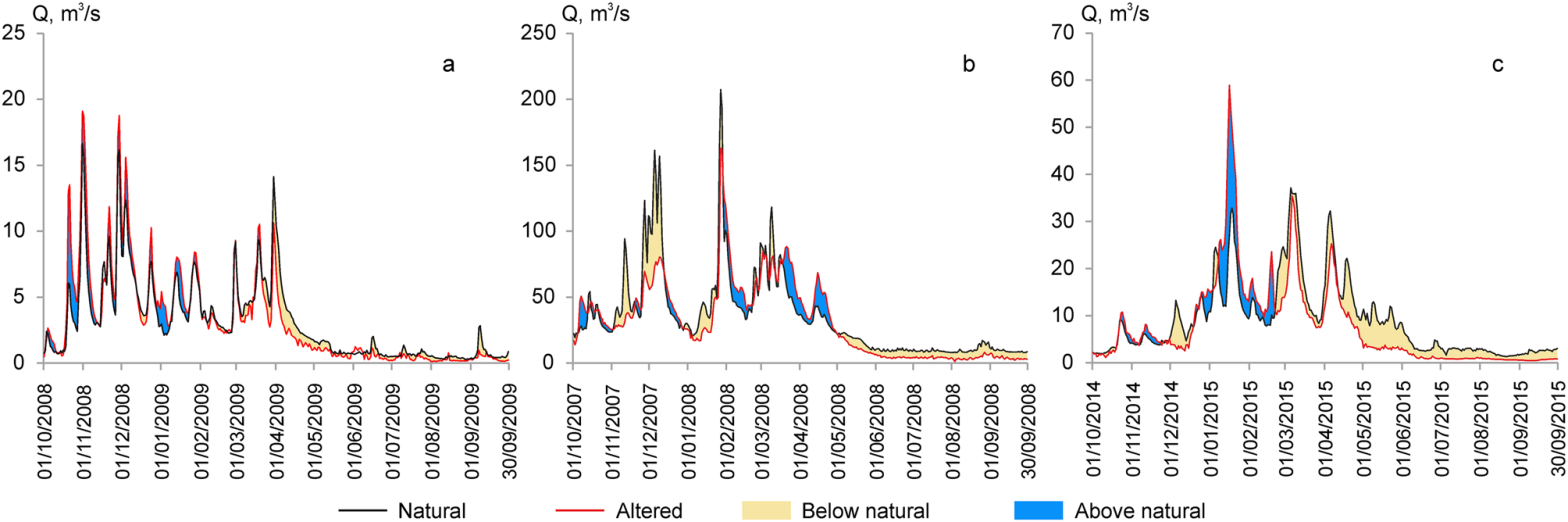
A comparison of hydrographs of natural and altered conditions during the dry years in the Bartuva River below Skuodas HPP (**a**), the Venta River below Kuodžiai HPP (**b**), and the Mūša River below Dvariukai HPP (**c**).

The hydrographs of studied sites, both compiled based on actual measurements at altered conditions and restored for natural hydrological regimes (without HPP activity), differ in the pattern and amount of annual discharge (Fig. 2). Redistribution of the flow stored upstream of the dams along the year was the main effect of HPPs on rivers discharge. In general, dams reduced low flow discharge in the spring/summer period and increased the flow rate after storing water during high flows. The related reduction of discharges during the warm season requires special focus and a more detailed analysis of this phenomenon, since it creates the most vulnerable conditions for the fish habitats.

**Figure 3:**
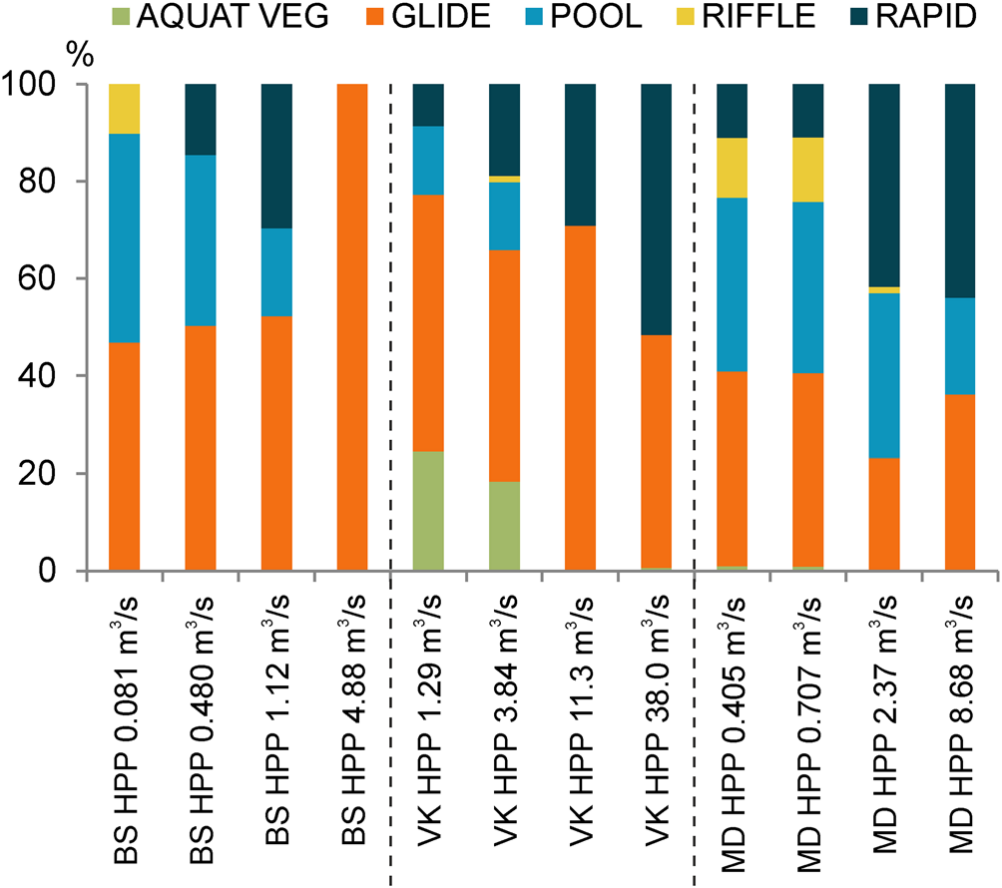
Changes in the relative area of different hydromorphological units according to different discharge conditions (close to Q_30_min_, Q_30_ave_, Q_30_max_, Q_annual_mean_) downstream HPPs of Bartuva-Skuodas (BS), Venta-Kuodžiai (VK), and Mūša-Dvariukai (MD).

**Figure 4:**
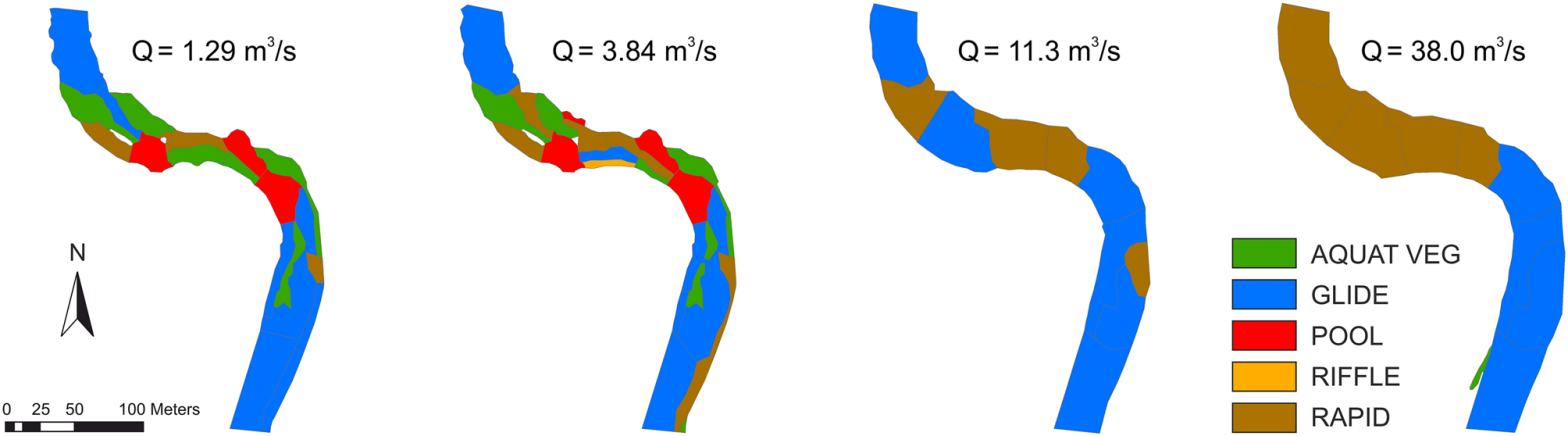
Distribution of hydromorphological units according to different discharge situations at the case study of Venta-Kuodžiai.

Based on measurements conducted at four different discharge conditions (close to Q_30_min_, Q_30_ave_, Q_30_max_, and Q_annual_mean_) in each site of the selected rivers, the relative area of hydromorphological units (HMUs) changed considerably together with the flow (Fig. 3). Diversity as well as the total number of HMUs per studied river stretch in general was higher at lower discharges, as it is illustrated in Fig. 4 for the Venta River. In total, 28 HMUs were mapped at low flow average conditions in the Venta River. Along with the increase of discharges, the HMUs got more homogenous and just a two types of them (glide and rapid) became dominant. Similar results were obtained for the rest of studied rivers, but the distribution of HMUs varied between other selected case studies, especially in changes of their area (see Supplementary Figs. S1 and S2 online). In the Bartuva River downstream Skuodas HPP, the HMU types such as glide and pool were the most dominant at the discharges of minimum low flow and average low flow. Due to increase in flow speeds, the riffle-type HMUs, which were present at the minimum discharge, at the average low flow changed into the rapid-type HMUs, whereas at the annual mean discharge hydromorphology unified and became of the type of glide. In the case of the Mūša River, the minimum low flow and the average situations differed only in hydrological parameters (discharge, depth and velocity) but the spatial distribution of HMUs was quite similar. During higher water discharges, the area of rapid-type HMUs strongly increased, while the area of riffle-type HMUs decreased and disappeared at the discharge of the annual mean.

### Conditional habitat suitability criteria

Conditional habitat suitability criteria (CHSC), developed for selected species based on the analysis of available data, are provided in Table 2. Testing with calibration dataset (85 sites), CHSC correctly predicted the presence of species in 67–75% of sites. Using abundance criteria, CHSC correctly predicted the presence of species in 76–90% of sites, with species abundance being greater than the median in 51–62% of them. Species were absent in 75–100% of sites where at least one of 4 main variables did not meet presence criteria.

**Table 2:** Conditional habitat suitability criteria for selected species.

Species	Water depth (m)	Water velocity (ms^−1^)	Substrate (any of listed types)	Cover (any of listed types)
Schneider	> 0.45 (> 40%) > 0.6* (> 40%)	0.15–1.0 (> 30%) 0.3–0.9* (> 40%)	Mesolithal, microlithal*, acal*, psammal* (> 70%; > 90%*)	Not applicable
Dace	> 0.3 (> 30%) 0.45–0.9* (> 30%)	< 0.9 (> 30%) 0.15–0.6* (> 30%)	Mesolithal*, microlithal*, acal*, psammal (> 70%; > 80%*)	Woody debris, boulders
Roach	> 0.3 (> 40%) > 0.6* (> 50%)	< 0.75 (> 30%) < 0.45* (> 30%)	Not applicable	Emerged vegetation, submerged vegetation
Vimba	0.3–0.9	0.15–0.6	Mesolithal, microlithal, acal	Submerged vegetation

Star symbol (*) indicates abundance criteria. The percentage in parentheses indicates the minimum area of the HMU that must meet the criteria.

Validation using data collected in HMUs of natural river stretches revealed similar results (Table 3). CHSC correctly predicted the presence of species in 67–81% of sites. Using abundance criteria, CHSC were correct in predicting the presence of species in 81–92% of sites, with species abundance being greater than the median in 50–69% of sites. Species were absent in 69–100% of sites where at least one variable did not meet presence criteria. The values of true skill statistic (TSS) were > 0 for all species, which confirms that performance of CHSC is not random. The validation results in natural rivers met the criteria for representativeness of CHSC and confirmed that CHSC can be used for modelling.

**Table 3:** Proportion of correctly predicted presence, abundance and absence, as well as overall proportion of correctly classified instances (*CCL*), sensitivity (*Sn*), specificity (*Sp*) and true skill statistic (*TSS*) of CHSC in natural rivers and river stretches below HPPs.

Species	Nb. of HMUs	Presence	Abundance		ABSENCE	*CCL*	*Sn*	*Sp*	*TSS*
			Present	Abundant					
	**Natural rivers**								
Schneider	42	0.76 (n-25)	0.85 (n-20)	0.55	0.82 (n-17)	0.79	0.83	0.70	0.53
Dace	42	0.74 (n-23)	0.81 (n-16)	0.50	0.74 (n-19)	0.74	0.77	0.70	0.47
Roach	42	0.73 (n-30)	0.90 (20)	0.65	1.00 (n-12)	0.81	0.71	0.60	0.31
Vimba	13	0.67 (n-9)			0.75 (n-4)	0.69	0.60	0.50	0.10
	**Stretches below HPPs**								
Schneider	47	0.56 (n-9)	0.67 (n-6)	0.33	0.89 (n-38)	0.83	0.56	0.89	0.45
Dace	47	0.56 (n-27)	0.57 (n-7)	0.29	0.75 (n-20)	0.64	0.75	0.56	0.31
Roach	47	0.85 (n-27)	0.75 (n-8)	0.38	0.55 (n-20)	0.72	0.72	0.73	0.45
Vimba	14	0.50 (n-6)			0.50 (n-8)	0.50	0.43	0.57	0.00

Numbers in brackets denote the number of HMUs in which species had to be present, abundant or absent based on CHSC.

In the HMUs of the river stretches below studied HPPs, the mismatch between predicted and observed occurrence and, particularly, abundance of species was greater compared to natural rivers (Table 3), probably due to disturbance of fish distribution caused by flow alteration. Only the presence of roach in the river stretches below HPPs was predicted with a higher accuracy compared to that in the natural river stretches. The absence of dace and schneider was predicted with a similar precision as in the natural rivers, but roach and vimba were also present in 45–50% of HMUs where they were supposed to be absent according to CHSC.

### Impact of HPPs on fish

In total, 18 fish species were found in the studied river stretches below HPPs under conditions close to average low flow, the roach being among the dominant species in all 3 rivers (Table 4). The share of the remaining modelled species was low below HPPs, with the exception of dace in the Bartuva River. Schneider was not found at all in the Mūša River, while the migration of vimba to this river is limited by an artificial obstacle.

**Table 4:** Fish species composition and relative abundance per whole sampled area in the studied rivers stretches below HPPs.

	*Alburnoides bipunctatus*	*Alburnus alburnus*	*Barbatulus barbatulus*	*Blicca bjoerkna*	*Cobitis taenia*	*Cottus gobio*	*Esox lucius*	*Gasterosteus aculeatus*	*Gobio gobio*	*Leuciscus leuciscus*	*Lota lota*	*Perca fluviatilis*	*Phoxinus phoxinus*	*Pungitius pungitius*	*Rhodeus sericeus*	*Rutilus rutilus*	*Squalius cephalus*	*Vimba vimba*
Bartuva	0.2	6.3	5.7		3.7		0.1		25.2	15.8	0.1	1.7	14.8		4.4	20.8	1.4	
Venta	4.7	15.0	6.6	0.1		0.6	0.0	0.8	14.8	1.0		0.6	3.5		1.6	48.0	1.3	1.5
Mūša		1.3	35.1		1.7		2.8	2.5	5.1	1.5		17.7		0.2		27.9	4.2	

**Figure 5:**
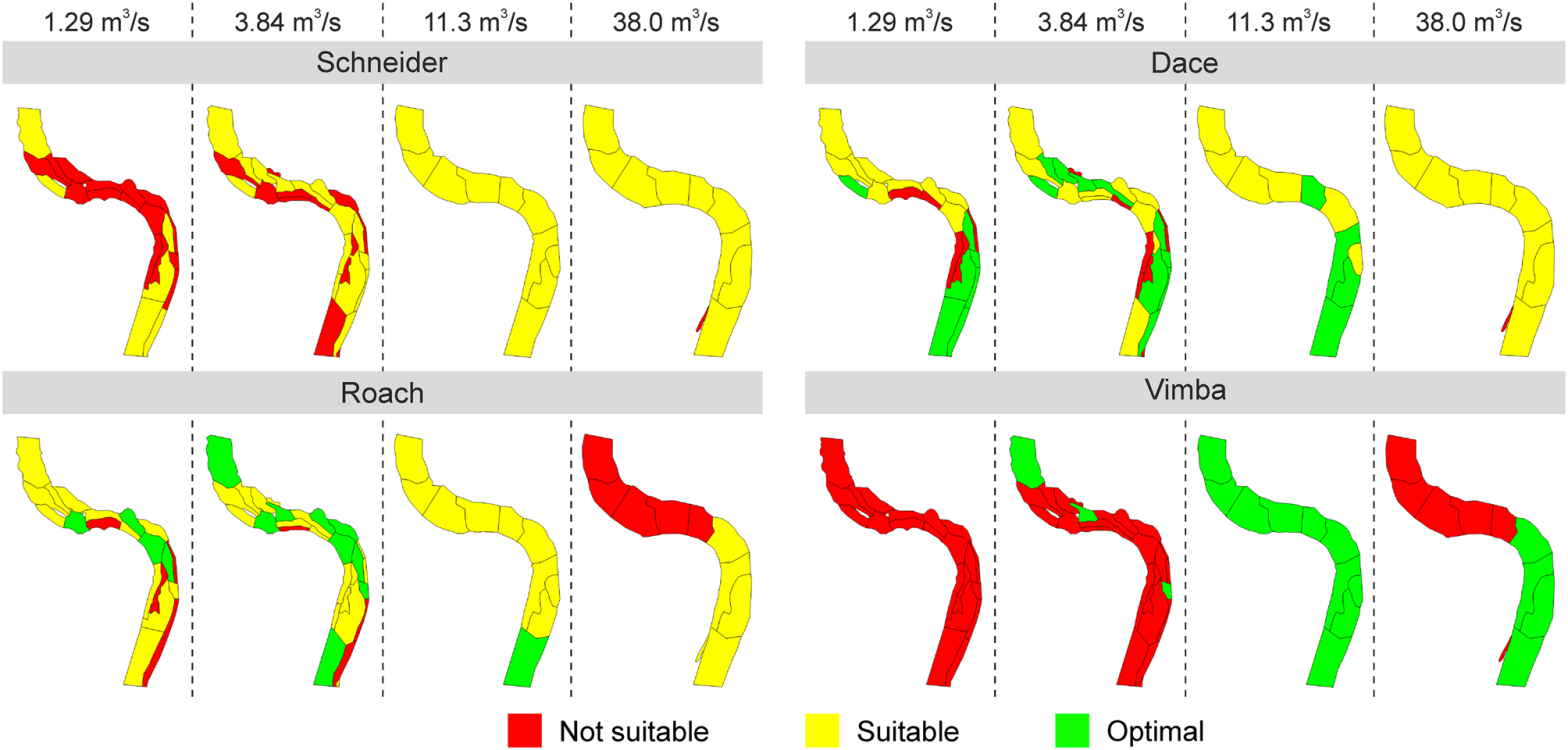
Habitat suitability maps of four fish species in Venta-Kuodžiai case study at four different discharge (m^3^/s) situations.

The habitat suitability maps composed using SimStream software showed that at the minimum of low flow discharge the studied stretches became unsuitable for schneider in the Mūša River below Dvariukai HPP and only partly suitable in the Bartuva and Venta. The same was valid for vimba in the Venta River downstream Kuodžiai HPP, as it is shown in Fig. 5 (for the Mūša and Bartuva rivers see Supplementary Figs. S3 and S4 online). At the minimum of low flow discharge, the studied stretch was not suitable for adult vimba and only partly suitable for schneider, but was mostly suitable and optimal for dace and roach. Only a few of the HMUs were not suitable for the latter two species. At higher flows, the area of habitats suitable for vimba and schneider increased significantly. The area optimal for dace and roach, on the opposite, was present at the minimum low flow, while at high flow it transformed to suitable polygons.

**Figure 6:**
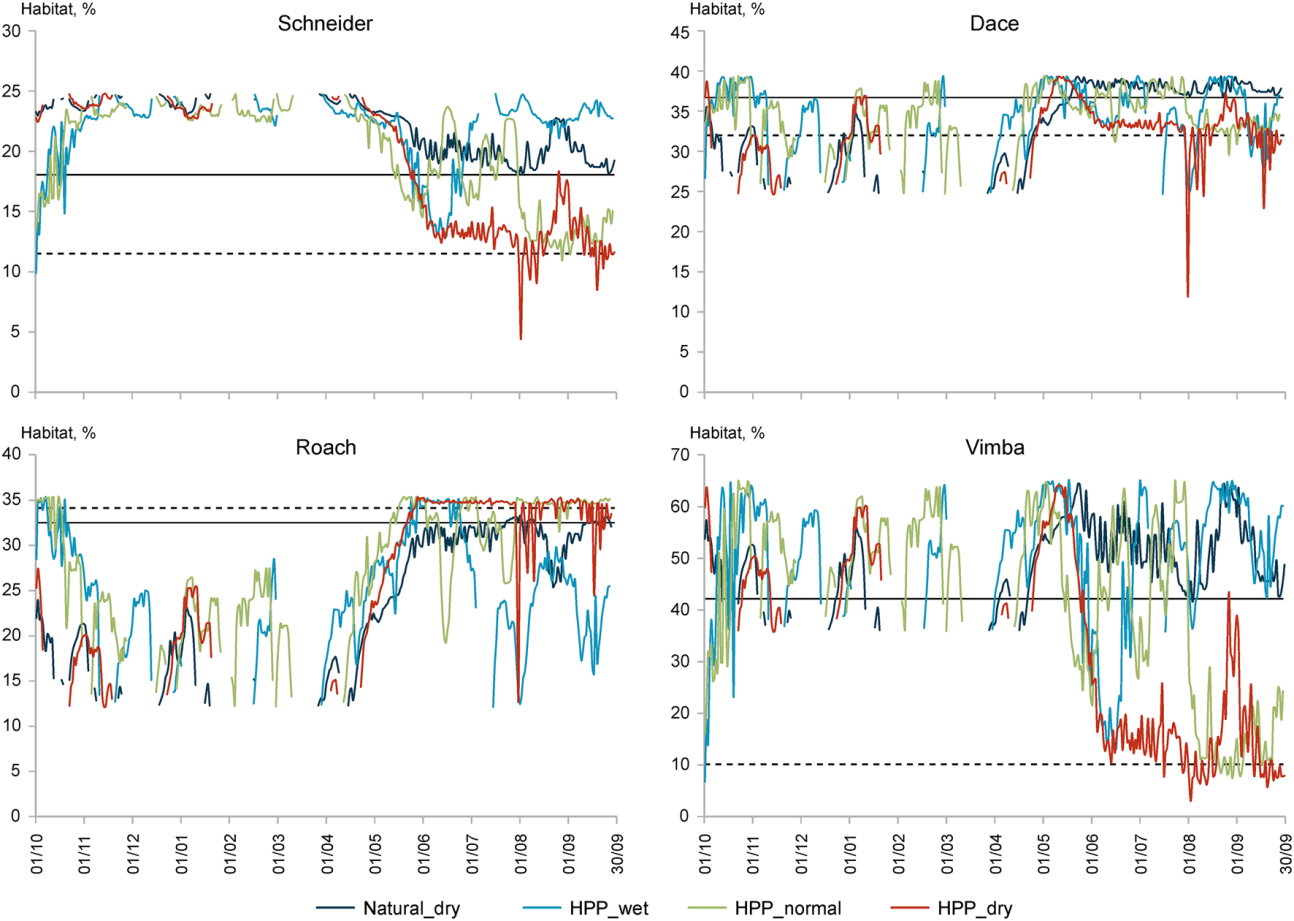
Time series of the suitable habitat area for the different fish species in the Venta River below Kuodžiai HPP for 12 months at natural conditions in a dry year and when HPP operates in a wet, normal, and dry year. The vertical axis represents habitat area (% of channel). The horizontal solid line indicates the habitat area at a Q_97_ discharge at natural conditions in a dry year, and the dashed line indicates the average area of the available habitat in July–August, when HPP operates in a dry year.

The time series of a suitable habitat that can be used by species over 12 months indicate that when HPPs operate in a dry year, the area of a suitable habitat deviates significantly from that at reference conditions (Fig. 6; also see Supplementary Figs. S5 and S6 online). A comparison of the cumulative duration of events, when the area of a suitable habitat falls below the threshold area (which is available for fish at reference conditions at Q_97_ in a dry year) at reference and altered conditions shows that the number of stress days significantly increases for the majority of simulated fish species under the operation of hydropower plants, especially in a dry year (Table 5). In the Bartuva and Mūša rivers, operation of HPPs in dry years had the strongest negative impact on the temporal availability of the habitat for the schneider, and in the Venta River such impact was even stronger for the vimba. In the Mūša River below Dvariukai, the habitat suitable for schneider in this section of the river generally became temporarily unavailable (ITH = 0). The HPPs had the least impact on the habitat of roach. In the Venta, the largest among the studied rivers, the area of the habitat suitable for roach even increased under operation of the HPP in a dry year (Fig. 6).

**Table 5:** The relative habitat area (% of river channel) of the modelled fish species at a discharge of Q_97_ in a dry year at natural conditions (S% at Q_97__reference_dry) and relative increase (in %) in the cumulative duration of stress days (stress days alteration; SDA) when the area of habitat falls below this threshold when HPPs function in a wet (SDA_altered_wet), normal (SDA_altered_normal) and dry (SDA_altered_dry) year.

River	Metric	Roach	Dace	Schneider	Vimba
Venta	S% at Q_97_ natural_dry	32	38	19	44
	SDA_ altered_wet	0 (1.00)	3 (0.99)	0 (1.00)	0 (1.00)
	SDA_altered_normal	16 (0.94)	65 (0.78)	67 (0.78)	139 (0.59)
	SDA_altered_dry	0 (1.00)	82 (0.73)	264 (0.37)	407 (0.21)
Mūša	S% at Q_97_ natural_dry	28	13	5	
	SDA_ altered_wet	12 (0.96)	133 (0.60)	124 (0.62)	
	SDA_altered_normal	23 (0.92)	162 (0.54)	144 (0.58)	
	SDA_altered_dry	209 (0.45)	559 (0.12)	645 (0.00)	
Bartuva	S% at Q_97_ natural_dry	15	10	7	
	SDA_ altered_wet	0 (1.00)	20 (0.93)	0 (1.00)	
	SDA_altered_normal	21 (0.92)	38 (0.87)	26 (0.91)	
	SDA_altered_dry	150 (0.57)	176 (0.51)	221 (0.43)	

The value of the index of temporal habitat availability (ITH) is given in brackets.

**Figure 7:**
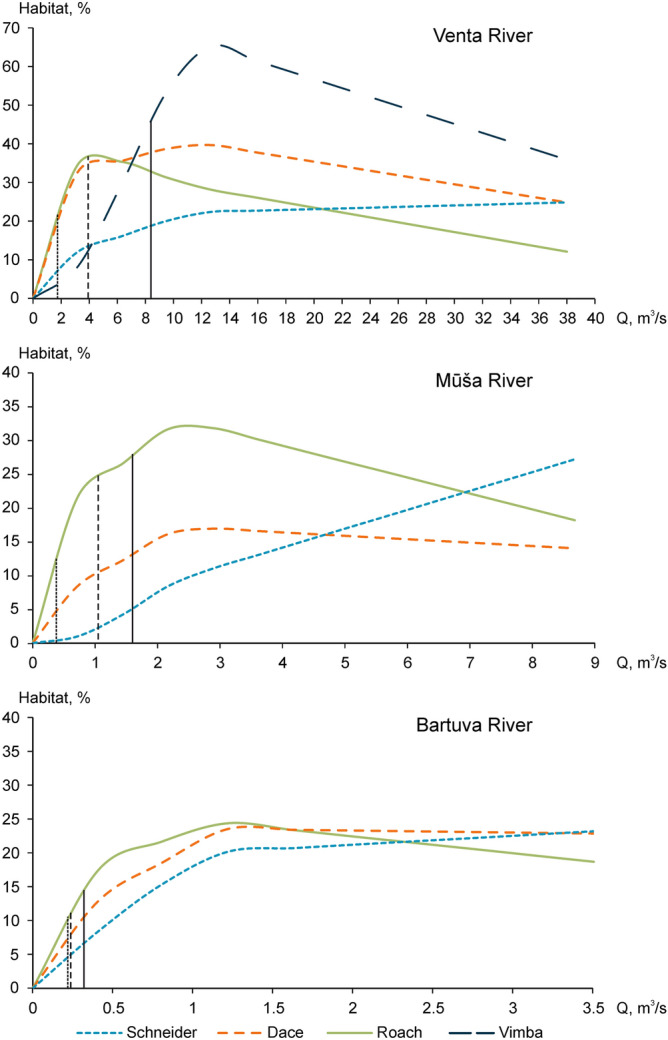
Habitat–flow rating curves for modelled fish species. The vertical axis represents habitat area (% of channel), and horizontal axis represents discharge (m^3^/s). The vertical solid line intersects the area of the habitat which is available for fish at Q_97_ at natural conditions in a dry year, the dashed line intersects the average area that can effectively be used by fish in July–August during the operation of HPPs in a dry year, and the dotted line indicates the area of the habitat which is present at Q_env_.

The modelled area of a suitable habitat that is temporarily available when HPPs operate in a dry year during the period of the least flow (July–August) approaches the area that is present at Q_env_ (Fig. 7). Compared with the maximum area of a suitable habitat that is available for fish at a certain (optimal) flow at reference conditions, the area at Q_env_ corresponds to 0–29% of the habitat area that is present at the optimal flow for schneider, 7% for vimba (in the Venta), 30–44% for dace and 50–80% for roach. Based on the results of habitat modelling at different flows, at a discharge of Q_env_, the relative area of the habitat suitable for vimba decreases to 4% in the Venta River, and the habitat suitable for schneider is absent in the Mūša River, with only a small area remaining in the Bartuva River. However, Q_env_ still guarantees relatively large areas of the habitat suitable for roach in all modelled rivers and for dace in the Venta and Bartuva rivers.

A general comparison of the relative abundance of the fish species typical of Lithuanian cyprinid rivers (species with a frequency of occurrence > 50%) in natural river stretches and those located below HPPs showed that the relative abundance of roach, bleak *Alburnus alburnus* and perch *Perca fluviatilis* was significantly higher in the stretches below HPPs. The relative abundance of schneider, on the contrary, was significantly lower in the stretches below HPPs, while the differences in the relative abundance of the rest of species were insignificant (Mann–Whitney U test) (Table 6).

**Table 6:** Relative abundance (mean ± SD) of common fish species (frequency of occurrence > 50%) in the river stretches with natural flow (n-42) and below HPPs (n-20) and the significance of their differences (Mann–Whitney U test).

	Natural	Below HPPs	Z	p-level
*Alburnoides bipunctatus*	19.1 ± 19.2	9.7 ± 15.9	2.42	0.015
*Alburnus alburnus*	4.3 ± 8.0	13.1 ± 15.1	− 2.61	0.009
*Barbatula barbatula*	10.2 ± 12.3	6.0 ± 8.6	1.76	0.077
*Cottus gobio*	7.4 ± 9.3	3.5 ± 4.7	1.85	0.063
*Esox lucius*	1.0 ± 1.2	1.8 ± 2.0	− 1.55	0.121
*Gobio gobio*	9.2 ± 7.9	9.4 ± 8.7	− 0.01	0.994
*Leuciscus leuciscus*	3.4 ± 6.4	5.6 ± 10.9	− 0.69	0.489
*Perca fluviatilis*	2.0 ± 6.7	5.0 ± 9.0	− 3.70	0.000
*Phoxinus phoxinus*	12.3 ± 9.7	7.2 ± 8.1	1.94	0.052
*Rutilus rutilus*	8.7 ± 9.7	26.5 ± 18.1	− 4.50	0.000
*Squalius cephalus*	5.2 ± 8.0	7.5 ± 11.5	− 1.69	0.092

## Discussion

In general, fish are resistant to short-term extreme environmental changes, and the capacity to cope with these changes is highly dependent on habitat heterogeneity^38,39^. However, the main factor determining the significance of the effects of extremes on fish is their duration^40^. In the most of Lithuanian unregulated rivers, an extreme decrease of the discharge to Q_env_ (i.e. probability of 95%) is observed on average once per 20 year. The frequency of discharges less than Q_env_ of the investigated rivers (the Mūša, Venta and Bartuva) under natural and altered conditions for the period after construction of HPPs (2001–2015) was compared. There was no fixed discharge below Q_env_ under natural river conditions (in reconstructed discharge time series without activity of HPPs). In regulated rivers with hydropower plants, the frequency of discharges below Q_env_ increased from 2.0% (Venta–Leckava WGS) to 11.5% (Bartuva–Skuodas WGS) during the dry period (May–September). This means that conditions become close to critical for fish over a long period of time. Based on the simulation results, when HPPs function in a dry years and discharge approaches Q_env_, the schneider temporarily loses most of the suitable habitats in all the studied river stretches. The same applies to the vimba in the Venta River and dace in the Mūša River. Meanwhile, Q_97_ discharge, which is present for a short period under reference conditions in a dry year, leads to a much smaller reduction in the area of the habitat. All this may be an explanation of why the schneider was not found at all in the Mūša River below the Dvariukai HPP (in a dry year, the modelled area of a suitable habitat under HPP functioning is close to zero), and only single individuals were found in the Bartuva River.

The simulation results also provide an explanation for the cases of mass death of fish that were observed in the Venta River below the Kuodžiai HPP in 2018 and, again, in 2019. In both years, the mass death of fish occurred during the period of the least discharge, in late July–early September, in a stretch of the river below the dam. According to the official reports of the Environmental Protection Department (the Ministry of Environment of the Republic of Lithuania), in 2018, the HPP released discharge which was lower than Q_env_, and this led to the death of various species of fish. However, in 2019, the data of the gauging station downstream the HPP confirmed that Q_env_ was guaranteed. Despite this, 260 dead vimba individuals were found in a 1.3-km stretch of the river below the HPP. It is known that reduced flow that occurs downstream of the HPPs can lead to an increase in aquatic vegetation^41–43^. This happens during the warm period, with the high water temperatures and oxygen consumption, as well as a reduced ability of water to carry oxygen. If in river stretches with dense vegetation downstream of HPP a decrease in discharge occurs at night with an increased oxygen consumption, this can lead to a more significant decrease in the oxygen content in the water compared to conditions at natural discharge. Among the studied rivers, relatively large areas covered with aquatic vegetation were present only in the Venta River. Therefore, it is likely that the vimba died out due to decrease in oxygen, which occurred due to a decrease in discharge at night. Smaller and less oxygen sensitive fish species probably survived in these conditions.

Unlike other simulated fish species, the impact of HPPs on the habitat of roach is much lower. Among the studied rivers, the operation of a HPP in the largest river Venta provides even better conditions for roach than under reference conditions. Based on CHSC, roach is the only species among those covered by this study, which prefers habitats with either submerged or emerged vegetation. The presence of relatively large areas of such habitats may be favourable for roach, provided that the water depth is sufficient and the current velocity is not too high. Due to the decrease in the current velocity under Q_env_, the relative habitat area suitable for roach remains significantly larger in other studied rivers too, compared to those for other modelled fish species. This partially explains why in the rivers of Lithuania, in stretches below HPPs, roach, as well as other less specialized eurytopic fish species, such as bleak or perch^29^, become dominant. The relative abundance of these species below HPPs is much higher, while that of schneider, on the contrary, is lower than the unregulated river stretches. Testing of CHSC in the natural river stretches and below the HPP also showed that roach was present in 45% of HMUs below the HPPs in which, according to CHSC, it should be absent. Meanwhile, in natural stretches, the absence of roach was predicted correctly in all cases. Although this was not analysed in this study, it is likely that in the river stretches below the HPPs not only relative but also absolute abundance of roach increases, while the numbers of intolerant species decrease. Reduced interspecific competition may open up additional opportunities for eurytopic roach, which is able to cope with a wider range of environmental conditions than more specialized rheophilic species can. Increased intraspecific competition could also force the roach to change their habitat^44,45^.

Both the results of the fish habitat modelling and the actual data confirm that at the low flow conditions the impact of low-head HPPs on the area of the habitat suitable for certain fish species becomes significant. The trend towards a significant increase in the proportion of eurytopic roach, perch and bleak below the HPPs, which are all resistant to habitat degradation^31^, also suggests that low-head HPPs may cause changes in ecological status. The tolerant species ecological guild is used in many of fish-based methods of Central-Baltic European countries as a metric that increases with degradation of a river^46^. Taking into account the projected decrease in the flow of Lithuanian rivers in the future^12^, the impact of HPPs on fish can become even more adverse if the regulation of HPPs operation based on the current Q_env_ definition will remain unchanged.

According to Parasiewicz et al.^47^, in the lowland rivers of neighbouring Poland, the e-flow during the rearing and growing bioperiod (May–September) corresponds to 0.93 or 0.95 of the average low flow. Theoretically, this may be applicable to the lowland rivers of Lithuania with a comparable seasonal distribution of discharge. When applying those coefficients, the e-flow for May–September is estimated to be 50% higher than the Q_env_, which is currently used throughout the year. For all studied rivers, this e-flow approximately corresponds to 0.3 of the optimal flow modelled for vimba and schneider. The e-flow proposed by Parasiewicz et al.^47^ for lowland rivers in the bioperiod of rearing and growing, is very close to the average low flow, which naturally occurs more often, and the fish assemblages should be more adapted to it. Consequently, the use of an average low flow would be a much better alternative to the current Q_env_, which has never been proven environmentally friendly.

The present research focused on assessing the impact of low-head HPPs based on the modelling of the relationship between habitat and water discharge at the low flow season, when HPPs impact is the most damaging^48^. The results of study confirm that the correct establishment of e-flow is crucial in the environmentally friendly management of the run-of-river hydropower facilities. However, the summer low flows are expected to decrease, and the dry periods are likely to become more frequent in the future^12^. With limited water availability, ensuring energy production and suitable conditions for the maintenance of aquatic communities will be a challenge, as inflow into HPPs may become lower not only for the established e-flow but also for Q_env_. Nevertheless, a solution can always be found. For instance, many advanced turbines having a wide range of capacities and different designs (e.g., double regulated or cross-flow) are currently being developed^13^. These features could provide such important flexibility here, as well as suitability for a particular natural flow regime. Punys et al.^13^ suggest that by applying simple turbine operational measures—step-wise turbine start-up and shut-down together with varying their number and capacities during 24 h—sudden changes of river flow can be substantially alleviated. However, total avoidance of downstream hydrograph ramping is not possible without applying structural measures (involving physical constructions) for run-of-river projects with impoundments^13^. In any case, the balance between the operational needs of HPPs and the environment will have to be set considering the possible effects of climate change on river discharge.

## Supplementary Information

Supplementary Information.

## Data Availability

The data that support the findings of this study are available from the corresponding author upon reasonable request.
