# Supplementary material for: Impacts of low-head hydropower plants on cyprinid-dominated fish assemblages in Lithuanian rivers
Source: Sci Rep. 2020 Dec 10;10:21687. doi: 10.1038/s41598-020-78701-8 (PMC7728750; doi:10.1038/s41598-020-78701-8)
Supplement: Supplementary file 1 — Supplementary Information. [file 41598_2020_78701_MOESM1_ESM.pdf]

Impacts of low-head hydropower plants on cyprinid-dominated fish assemblages in Lithuanian rivers

Tomas Virbickas [1,*], Paolo Vezza [2], Jūratė Kriaučiūnienė [3], Vytautas Akstinas [3], Diana Šarauskienė [3], Andrius Steponėnas [1]

[1] Nature Research Centre, Akademijos 2, 08412 Vilnius, Lithuania

[2] Politecnico di Torino, Department of Environment, Land and Infrastructure Engineering, Corso Duca degli Abruzzi, 24, 10129 Torino, Italy

[3] Lithuanian Energy Institute, Breslaujos 3, 44403 Kaunas, Lithuania

* e-mail: tomas.virbickas@gamtc.lt

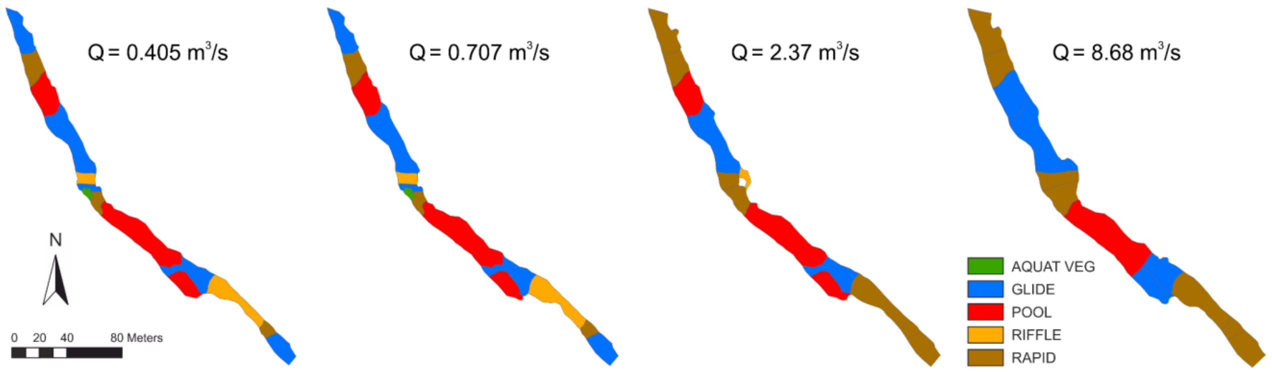

Supplementary Figure S1. Distribution of hydromorphological units according to different discharge situations at the case study of Mūša-Dvariukai

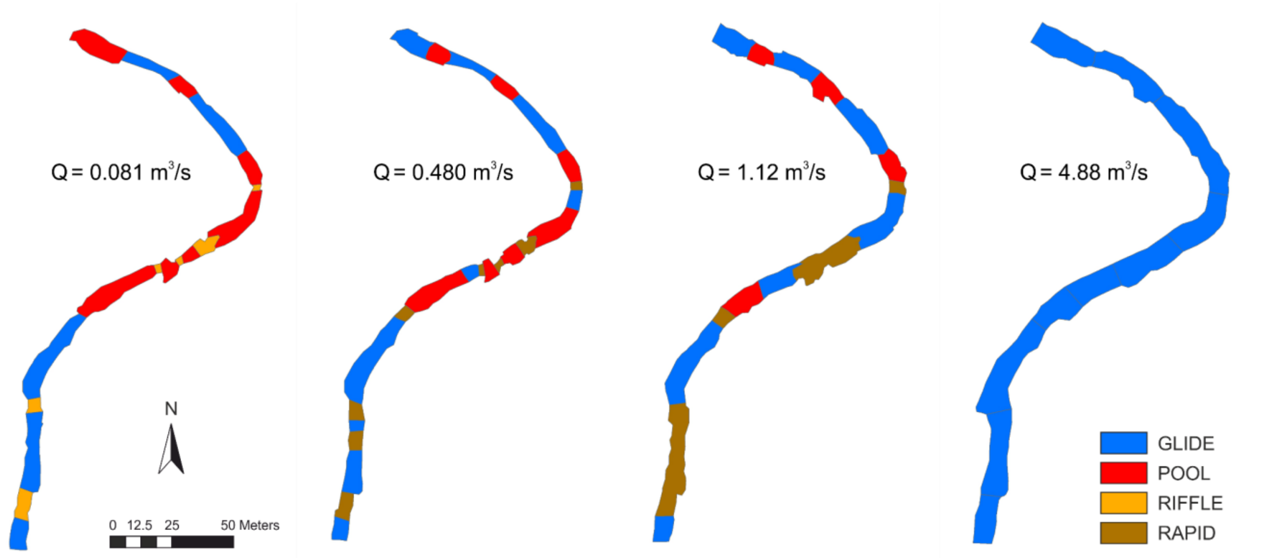

Supplementary Figure S2. Distribution of hydromorphological units according to different discharge situations at the case study of Bartuva-Skuodas

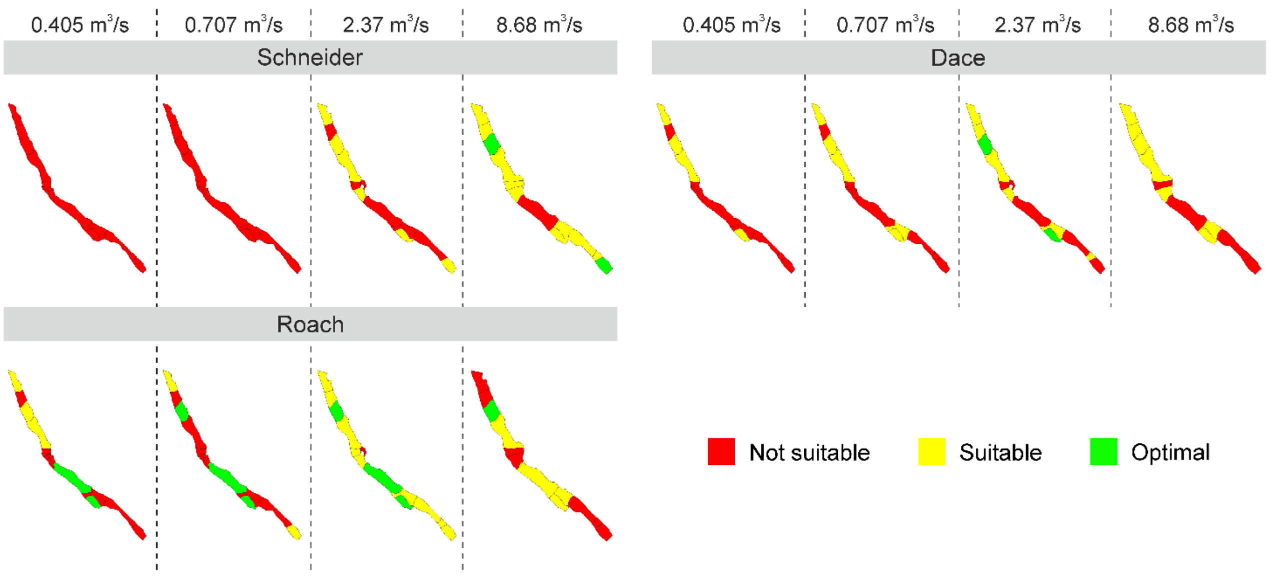

Supplementary Figure S3. Habitat suitability maps of three fish species in Mūša-Dvariukai case study at four different discharge (m^3/s) situations

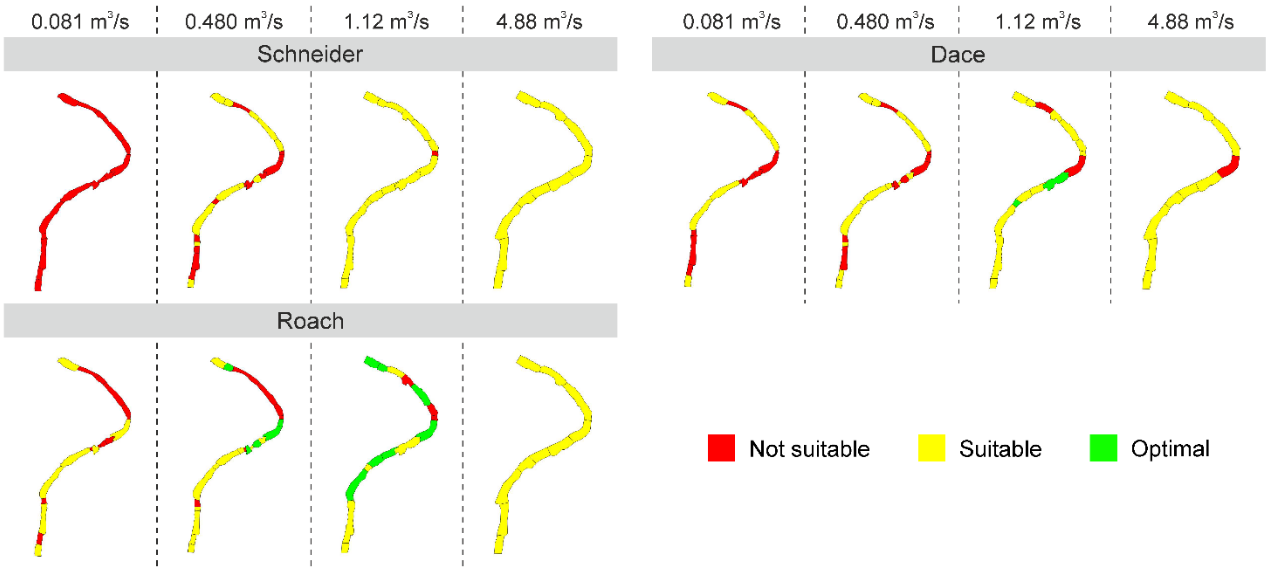

Supplementary Figure S4. Habitat suitability maps of three fish species in Bartuva-Skuodas case study at four different discharge (m^3/s) situations

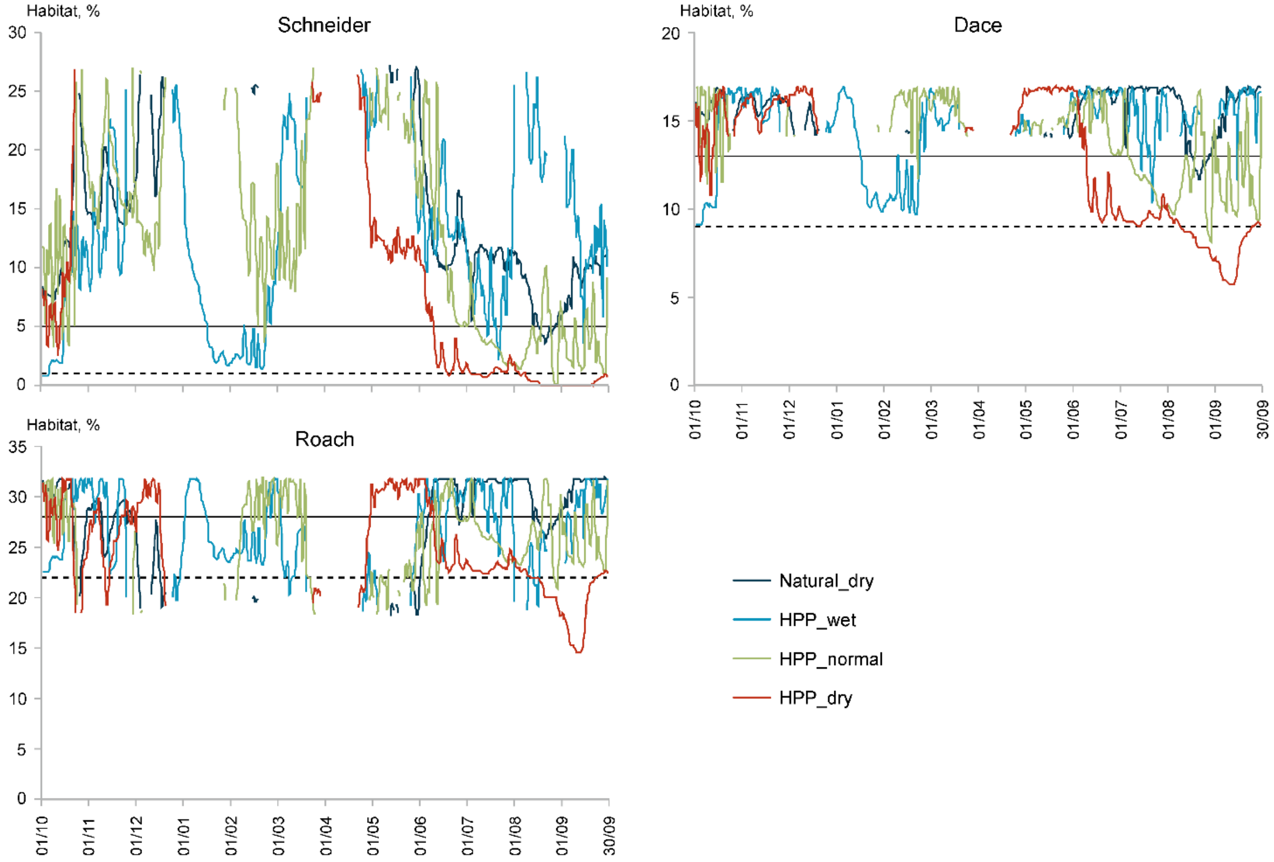

Supplementary Figure S5. Time series of the suitable habitat area for the different fish species in the Mūša River below Dvariukai HPP for 12 months at natural conditions in a dry year and when HPP operates in a wet, normal, and dry year. The vertical axis represents habitat area (% of channel). The horizontal solid line indicates the habitat area at a Q_{97} discharge at natural conditions in a dry year, and the dashed line indicates the average area of the available habitat in July – August, when HPP operates in a dry year

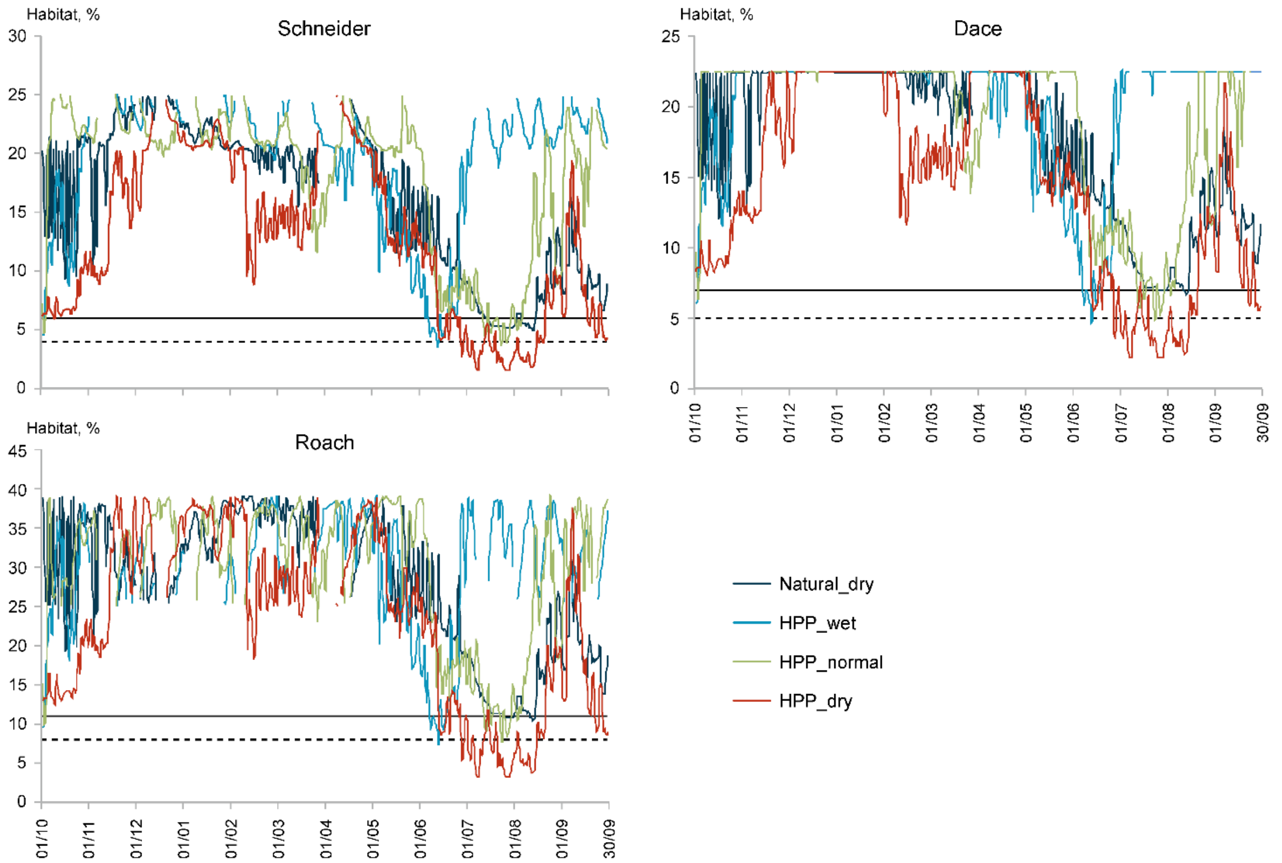

Supplementary Figure S6. Time series of the suitable habitat area for the different fish species in the Bartuva River below Skuodas HPP for 12 months at natural conditions in a dry year and when HPP operates in a wet, normal, and dry year. The vertical axis represents habitat area (% of channel). The horizontal solid line indicates the habitat area at a Q_{97} discharge at natural conditions in a dry year, and the dashed line indicates the average area of the available habitat in July – August, when HPP operates in a dry year
